# Changes in quantifiable breathing pattern components predict asthma control: an observational cross-sectional study

**DOI:** 10.1186/s40733-021-00071-3

**Published:** 2021-04-06

**Authors:** Panagiotis Sakkatos, Anne Bruton, Anna Barney

**Affiliations:** 1 School of Health Sciences, University of Southampton, Southampton, UK; 2 Institute for Sound and Vibration Research, University of Southampton, Southampton, UK

**Keywords:** Breathing Patterns, Within-Subject Variability, Physiological Marker, Asthma Control

## Abstract

**Background:**

Breathing pattern disorders are frequently reported in uncontrolled asthma. At present, this is primarily assessed by questionnaires, which are subjective. Objective measures of breathing pattern components may provide additional useful information about asthma control. This study examined whether respiratory timing parameters and thoracoabdominal (TA) motion measures could predict and classify levels of asthma control.

**Methods:**

One hundred twenty-two asthma patients at STEP 2- STEP 5 GINA asthma medication were enrolled. Asthma control was determined by the Asthma Control Questionnaire (ACQ7-item) and patients divided into ‘well controlled’ or ‘uncontrolled’ groups. Breathing pattern components (respiratory rate (RR), ratio of inspiration duration to expiration duration (Ti/Te), ratio of ribcage amplitude over abdominal amplitude during expiration phase (RCampe/ABampe), were measured using Structured Light Plethysmography (SLP) in a sitting position for 5-min. Breath-by-breath analysis was performed to extract mean values and within-subject variability (measured by the Coefficient of Variance (CoV%). Binary multiple logistic regression was used to test whether breathing pattern components are predictive of asthma control. A post-hoc analysis determined the discriminant accuracy of any statistically significant predictive model.

**Results:**

Fifty-nine out of 122 asthma patients had an ACQ7-item < 0.75 (well-controlled asthma) with the rest being uncontrolled (*n* = 63). The absolute mean values of breathing pattern components did not predict asthma control (R^2^ = 0.09) with only mean RR being a significant predictor (*p* < 0.01). The CoV% of the examined breathing components did predict asthma control (R^2^ = 0.45) with all predictors having significant odds ratios (*p* < 0.01). The ROC curve showed that cut-off points > 7.40% for the COV% of the RR, > 21.66% for the CoV% of Ti/Te and > 18.78% for the CoV% of RCampe/ABampe indicated uncontrolled asthma.

**Conclusion:**

The within-subject variability of timing parameters and TA motion can be used to predict asthma control. Higher breathing pattern variability was associated with uncontrolled asthma suggesting that irregular resting breathing can be an indicator of poor asthma control.

## Introduction

The goal of asthma management is to achieve optimal asthma control [1]. To assess asthma control, symptom questionnaires are currently used in clinical practice [2]. As this can be sometimes misleading due to their reliance on the patients’ perceptions, objective physiological markers, such as lung function, are commonly used alongside with symptoms questionnaires to ensure asthma progress [3]. To date, traditional lung function tests primarily provide information about airway calibre and lung volume whilst monitoring single forced expiratory maneuvers. On the other hand, this lacks monitoring of the natural behaviour of breathing causing ambiguity about the clinical significance of changes in tidal breathing in relation to different levels of asthma control.

Natural behaviour of breathing is assessed by quantifying breathing patterns with breathing pattern comprising components of volume, timing and thoracoabdominal (TA) movements [4]. Breathing pattern components, such as tidal volume (Vt), timing parameters (inspiration and expiration duration or their ratio, respiratory rate (RR)) and TA motion, can now be measured non-invasively over time without requiring patients’ cooperation compared to traditional lung function tests [5, 6]. To date, breathing pattern disorders (also known as dysfunctional breathing) are commonly reported in patients with uncontrolled asthma, even though their relationship (causal or coincidental) has not been clearly determined yet [7, 8]. The most commonly reported respiratory symptoms of dysfunctional breathing are predominant upper thoracic breathing, asynchrony between ribcage and abdominal motion, breathlessness, chest tightness, wheezing and deep sighing [9]. However, most of these have been described subjectively through clinicians’ observations or using symptom questionnaires, such as the Nijmegen Questionnaire (NQ) [10]. The use of the NQ in this way has been criticised due to its reliance on patients’ perceptions and the lack of incorporating direct measures of quantifiable breathing pattern components [11, 12].

Changes in a limited number of quantifiable breathing pattern components have been previously reported among asthma patients [13], but any relationship of them among different levels of asthma control have not been established yet. A positive weak correlation (r = 0.33) has been reported between TA asynchrony, as measured using Respiratory Inductive Plethysmography (RIP), and Asthma Control Questionnaire (ACQ7-item) [14]. Raoufy et al. [15] has also reported that within-subject variability of Vt and breath cycle duration as measured by the RIP, could differentiate uncontrolled asthma patients (*n* = 10) from patients with well-controlled asthma (n = 10) as determined by the presence of asthma symptoms. However, no firm conclusions have been drawn regarding the use of other multiple breathing pattern components, such as respiratory timing components or individual movements of TA area, to predict asthma control. Moreover, the optimal breathing pattern measures used to classify levels of asthma control have not been clearly specified yet.

Considering all the above, the study’s aim was to obtain measures of multiple quantifiable breathing pattern components, including both mean and within-subject variability measures, and determine whether changes in respiratory timing components and individual TA movements can be used to predict and classify levels of asthma control.

## Methods

This observational cross-sectional study recruited 122 adult asthma patients with a range of asthma severity from a difficult-to-treat outpatient clinic at the University Hospital Southampton and from staff and students at the University of Southampton. The sample size was determined based on the number of events per variable as proposed in [16]. This indicated that a sample size of 20 subjects per predictor in the regression model for each category of the binary outcome (ACQ questionnaire) could be expected to give a credible outcome for a logistic regression analysis. Individuals with a medical diagnosis of asthma without any other chronic respiratory disease or any upper respiratory tract infection on the day of data collection were eligible for this study. Levels of asthma control were determined by the ACQ7-item_,_ and cut-off points < 0.75 and > 1.50 were used to define well-controlled and uncontrolled asthma respectively. Asthma patients with partially-controlled asthma (ACQ7-item scores between 0.75 and 1.50) were not included in this study. All participants were between STEP 2 and STEP 5 asthma medication according to GINA guidelines [1].

After obtaining informed consent, participants’ demographic data and medication history were collected. Asthma medication data was used to determine asthma severity. Participants’ breathing pattern components were recorded during resting breathing in a seated position and then spirometry (Vitalograph) was performed to evaluate lung function.

Breathing pattern components were recorded using the Structured Light Plethysmography (SLP, Thora-3Di™, Pneumacare Ltd) according to manufacturers’ guidelines [17]. This is a non-invasive motion-analysis recording system. It comprises a contactless device which projects a grid pattern of light onto an individual’s chest wall covering the area between the clavicles and the umbilicus. The distortion of the grid pattern intersection points caused by the displacement of the anterior surface of the chest wall is recorded by two digital cameras. The two digital cameras are attached on the SLP which generates a time-varying output trace. The manufacturer’s own software did not allow direct breath-by-breath estimations of ribcage and abdominal amplitudes (RCampe and ABampe). Thus, an automatic peak detection algorithm written in Matlab code and used in our previous research [18] was used to obtain values of breathing pattern components during a breath by breath analysis of SLP’s output trace.

The automatic algorithm identified local minima and maxima of the inspiration phase for each breath cycle. The RR was defined as the number of complete breath cycles in one minute and the inspiratory/ expiratory phase ratio (Ti/Te) was defined as the proportionality between inspiratory and expiratory phases. The inspiratory time (Ti) was calculated as the time between a minimum in the sum SLP output trace and the next peak. The expiratory time (Te) was calculated as the time between a peak and the next minimum. The ribcage and abdominal amplitudes (RCampe and ABampe) were defined as the vertical distances between a trough and the next peak on the SLP’s output as derived from the different SLP’s traces used to record the motion of the ribcage and abdomen separately. The within-subject variability of the breathing pattern components was calculated as the Coefficient of Variance expressed as a percentage (CoV%).

The patients’ breathing pattern components were recorded for 5 min at the sitting position. The participants were requested to stay still and quiet during the whole recording procedure. This was to minimise external body movement artefacts on the SLP’s output trace as this could bias values of breathing pattern components during data extraction. When patients were ready to be recorded, they were falsely informed about the start of breathing pattern recording. The actual recording time started one minute after the initial notification. This was to eliminate any impact of the patients’ awareness on breathing pattern measurements whilst recording natural behavior of their breathing.

Descriptive statistics were used to summarise demographic data and lung function measurements Comparisons of the breathing pattern components between well-controlled and uncontrolled asthma groups were made using the Mann-Whitney U test (significance level *p* < 0.01) as normal distribution of the data was not found. Multiple binary logistic regression, using the forced method, was performed to predict uncontrolled asthma (ACQ7-item > 1.50). Two regression models were applied, one using absolute mean values of RR, Ti/Te and RCampe/ABampe as predictors. The other one involved the within-subject variability measures (Cov%). Both regression models met the assumption of multicollinearity (Variance Inflation Factor < 10). When all predictors of a regression model significantly predicted uncontrolled asthma, a post-hoc analysis using a Receiver Operating Characteristic curve (ROC) was used to identify cut-off points for changes in breathing pattern components distinguishing well-controlled and uncontrolled asthma.

## Results

One hundred twenty two adult asthma patients (75 females) were recruited and completed the study (mean age (sd) 44.75 years (15.98 years). Sixty-three participants had an ACQ score of > 1.5 (uncontrolled asthma), whereas 59 participants scored < 0.75 (well-controlled asthma). Thirty-three participants had mild asthma (STEP 2 on GINA asthma medication), with 29 of these being in the well-controlled group while the rest of them had moderate-to-severe asthma (STEP 3, 4 and 5 on GINA asthma medication). There were similar numbers of males and females in both groups (Table 1). Both groups also had similar average body mass index (BMI). Those in the uncontrolled asthma group had reduced average lung function compared to the well-controlled asthma group (Table 1).

**Table 1:** Demographic data and lung function measurements of asthma control groups

Variable	Well controlled asthma group (***n*** = 59)			Uncontrolled asthma group (***n*** = 63)		
Gender	23 males; 36 females			24 males; 39 females		
Asthma severity	29 mild; 30 moderate-to-severe			4 mild; 59 moderate-to-severe		
Age (years)	**μ**	**sd**	**95%CI**	**μ**	**sd**	**95%CI**
	41.20	17.78	36.83–45.58	48.06	14.56	44.40–51.73
BMI (kg/m^2^)	24.95	3.75	23.97–25.93	26.49	4.01	25.48–27.50
FEV_1predicted_ (%)	100.90	18.81	96.00–105.81	76.06	24.93	69.79–82.34
FEV_1_/FVC	81.91	9.44	79.45–84.37	74.49	15.28	70.64–78.34
PEF(l/min)	5.27	1.42	4.90–5.65	4.06	1.56	3.67–4.45

*μ* Mean value, *sd* standard deviation, *95%CI* 95% Confidence intervals; asthma control groups were determined by the ACQ7-item with scores < 0.75 and > 1.50 showing well-controlled and uncontrolled asthma respectively

Although those in the uncontrolled asthma group had significantly higher median RR than those in the well-controlled group, no significant differences were found for the other absolute mean values of breathing pattern components (Ti/Te and RCampe/ABampe) (Table 2). On the other hand, the within-subject variability measures (CoV%) of all the breathing pattern components were found to be significantly increased in the uncontrolled asthma group compared to the well-controlled group (Table 2).

**Table 2:** The differences in the breathing pattern components between asthma control groups

Breathing component	Well-controlled group (***n*** = 5 9)		Uncontrolled group (***n*** = 63)		Mann-Whitney U	***p*** (1-tailed)
	M^**a**^	Min-Max^**b**^	M	Min-Max		
RR (bpm)	14.92	7.09–21.05	17.16	7.40–32.02	1175	0.000*
Ti/Te	0.66	0.40–0.90	0.68	0.40–0.96	1689	0.385
RC_ampe_/AB_ampe_^c^	1.29	0.43–4.20	1.33	0.37–5.31	1798	0.729
CoV_RR_ (%)	4.79	0.00–23.02	11.73	0.00–29.71	655	0.000*
CoV_Ti/Te_ (%)	19.05	10.49–46.11	33.22	14.28–57.39	606	0.000*
CoV_RCampe_/_ABampe_^d^(%)	14.82	6.05–24.82	26.45	7.74–57.62	844	0.000*

^a^*M* median value

^b^Min-Max minimum and maximum values

^c^*RC*_*ampe*_*/AB*_*ampe*_ Ribcage to abdominal amplitude during expiration phase

^d^*CoV%*_*RCampe*_*/*_*ABampe*_ The within-individual variability of ribcage to abdominal amplitude during expiration phase

*significant result at *p* < 0.01

When mean values of RR, Ti/Te and RCampe/ABampe were entered into the regression model asthma control was not predictable with only the beta coefficient of RR being significantly greater than zero (Table 3). When within subject variability measures (CoV%) of breathing pattern components were entered into the model, a good fit was found (Table 4). This accounted for 45% of the variance in the ACQ7-item scores. The beta coefficients of the CoV% of all breathing pattern components were found to be significantly greater than zero suggesting that increased within-subject variability of RR, Ti/Te and RCampe/ABampe predicts uncontrolled asthma. A linear relationship was found between the CoV% of all breathing pattern components and the log of the ACQ7-item score with no more than 5% of the total cases being considered as influential cases (standardised residuals > 2) in the specific regression model.

**Table 3:** The regression model including mean values of breathing pattern components used to predict uncontrolled asthma

Predictors	B (SE)	95% CI for Odds Ratio			***p***
		Lower	Odds Ratio	Upper	
RR (bpm)	0.16 (0.05)	1.06	1.17	1.30	0.002*
Ti/Te	0.10 (1.79)	0.03	1.10	37.36	0.954
RC_ampexp_/AB_ampexp_^	0.07 (0.29)	0.61	1.07	1.88	0.812

B_0_ 0.07; R^2^ 0.09; R 0.12; −2LL 157.38

*starred sig. Value was found to be significant at p < 0.01

**Table 4:** The regression model including the CoV% of breathing pattern components used to predict uncontrolled asthma

Predictors	B (SE)	95% CI for Odds Ratio			***p***
		Lower	Odds Ratio	Upper	
CoV_RR_(%)	0.15 (0.05)	1.05	1.16	1.29	0.000*
CoV_Ti/Te_(%)	0.10 (0.03)	1.04	1.11	1.18	0.001*
CoV_RCampexp_/AB_ampexp_(%)	0.09 (0.05)	1.05	1.11	1.17	0.005*

B_0_ 0.07; R^2^ 0.45; R 0.59; −2LL: 96.87

*starred values were significant results at *p* < 0.01

**Fig. 1:**
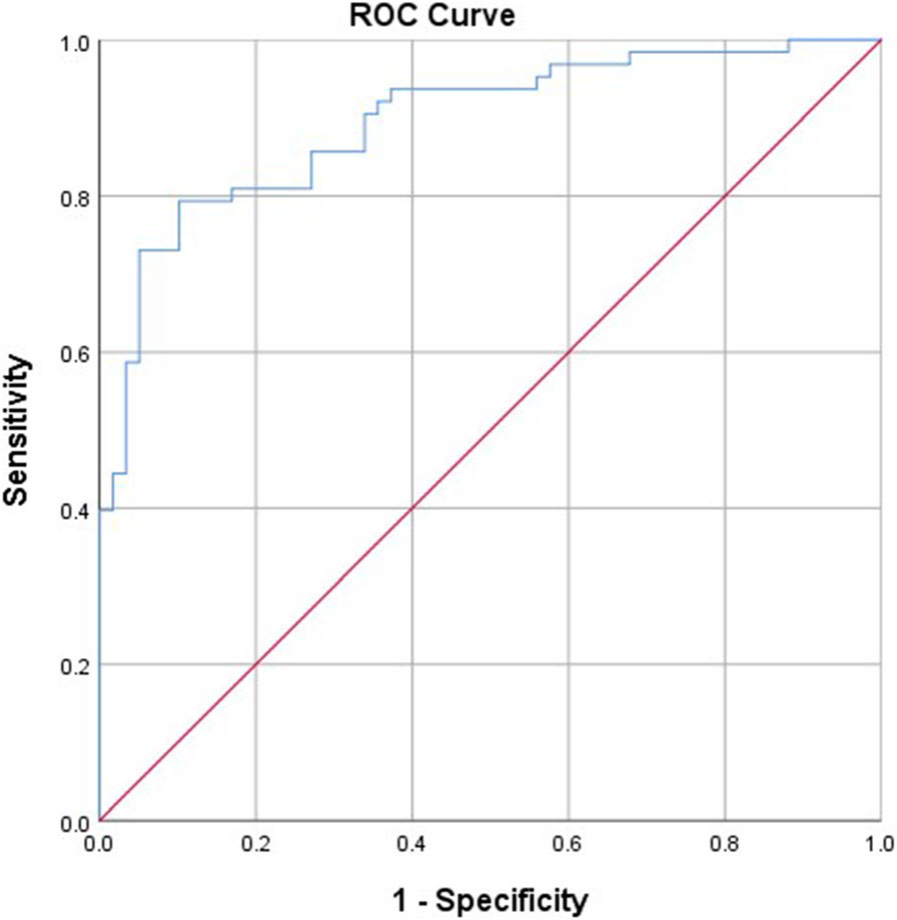
The ROC curve of the regression model including the CoV% of the examined breathing pattern components

**Fig. 2:**
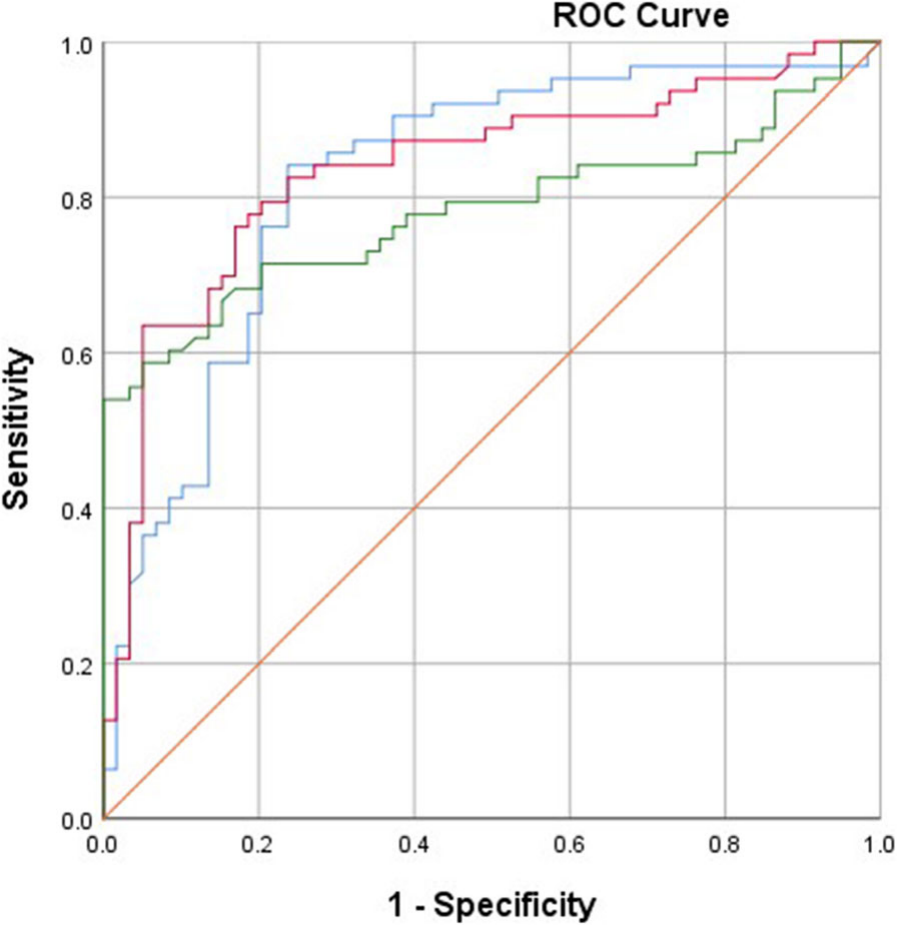
The different ROC curves for the CoV% of RR (blue line), Ti/Te (red line) and RCampe/ABampe (green line)

A post-hoc analysis showed that a regression model including the CoV% of breathing pattern components correctly classified 53 out of 59 patients with ACQ7-item < 0.75. It also correctly classified 48 out of 63 patients with ACQ7-item > 1.50. The sensitivity and specificity of the regression model were estimated to be 77.94 and 88.88% respectively with the area under the ROC being 0.895 (95% C I [0.84, 0.95], sig 0.000, *p* < 0.01) (Fig. 1). Based on individual ROCs for the CoV% of individual breathing pattern components (Fig. 2), a cut-off point > 7.40% for the CoV% of the RR discriminated well-controlled from uncontrolled asthma. Optimal cut-off points for the CoV% of Ti/Te and RCampe/ABampe were estimated to be > 21.66% and > 18.96% respectively (Table 5).

**Table 5:** Optimal cut-off points for the CoV% of each breathing pattern component and estimates of the area under the curve (AUC)

Breathing component	Optimal cut-off point^**a**^	AUC	Std error	95% CI	***p***
CoV_RR_ (%)	> 7.40	0.824	0.039	0.747–0.900	0.000*
CoV_Ti/Te_ (%)	> 21.66	0.837	0.038	0.763–0.911	0.000*
CoV_RCampe/ABampe_ (%)	> 18.78	0.773	0.044	0.686–0.859	0.000*

^a^Optimal cut-off points were selected as the closest points from the left corner of the individual ROC curves for the CoV% of each breathing paramete

*significant result was defined at p < 0.01

## Discussion

The study aimed to examine whether respiratory timing parameters and/ or individual TA movements could predict and classify levels of asthma control. The within-subject variability of breathing pattern components, such as RR, Ti/Te and RCampe/ABampe, was found to predict asthma control, but their absolute mean values did not. Based on these findings, the within-subject variability of breathing pattern components is suggested as a better indicator of asthma control than their mean values when measured in a single occasion. This may be because the within-subject variability can efficiently reflect changes in the natural behaviour of tidal breathing occurred in relation to asthma control. The importance of measuring the natural behaviour of breathing patterns has been previously highlighted as this reflects better the adaptability of the respiratory system occurred during symptomatic periods of asthma [19].

On the other hand, the limited variance we found in the absolute mean values of Ti/Te and RCampe/ABampe may have biased the asthma control prediction. Although the RR was found to be a significant predictor of asthma control, there was a lack of a linear relationship between mean RR and asthma control. For example, increased RR were not always associated with uncontrolled asthma. Lack of asthma control prediction using mean values of the examined breathing pattern components may be attributed to the presence of study’s confounders previously reported in other cross-sectional observational study designs [20, 21]. Examples of such confounders could be a postural effect, the patients’ asthma complexity, the underlying patients’ anxiety levels, and an effect of rescue medication usage prior to breathing pattern measurements. Some of these, such as posture and emotions, have been clearly suggested to affect absolute mean values of breathing pattern measurements [20–22], but the impact of asthma complexity and medication usage on breathing patterns is not clear yet.

Respiratory rate can be affected by different factors, and so there was no clear separation between the well-controlled and controlled groups for this parameter in our study. Asthma patients frequently have co-existing anxiety which can have an impact on the RR [23]. There is also a relationship between asthma and obesity [24], and it is well known that BMI can have an impact on asthma control and timing components of breathing patterns [25]. Although levels of anxiety were not assessed in our study, our study’s individuals with raised RR and well-controlled asthma were obese (BMI > 30 kg/m^2^). The normal RR found in individuals of the uncontrolled asthma group is unexplained, but could be due to the effect of rescue medication on RR. The participants were asked to state whether they had taken any type of asthma medication prior to breathing pattern measurements. No attempt was made to control the participants’ use of medication, they were just advised to take their medications as normal. It was established that all individuals had taken their controller medication as prescribed, but that patients with normal RR and uncontrolled asthma had additionally used rescue medication before attending the recording session. However, the impact of either short-acting or long-acting asthma medication on quantifiable breathing pattern components (both absolute or variability measurements) has not yet been established.

In addition, Raoufy et al. [15] have previously reported that the within-subject variability of Vt and breath cycle duration can differentiate patients with well-controlled asthma from those with uncontrolled asthma. Our findings are in agreement with Raoufy et al.’s work despite methodological differences, such as the method used to determine asthma control (National Asthma Education and Prevention program vs ACQ7-item), the breathing pattern recording time (60 min vs 5 min), the recording posture (supine vs sitting) and the equipment used to monitor breathing patterns (SLP vs RIP) at rest.

The optimal time for recording variability within breathing pattern parameters is not known in the literature. We measured within-subject variability over 5 min and found this was sufficient for making significant predictions of asthma control using respiratory rate, proportionality of respiratory phases, and TA motion. To the best of authors’ knowledge, the study presented here also provides for a first time specific cut-off points for the within-subject variability of the breathing pattern components, which differentiated well-controlled from uncontrolled asthma. However, more research is required to confirm the accuracy of our results in the future.

In addition, the different posture selected in our study compared to Raoufy et al. [15] did not seem to have an impact on the ability of within-subject variability of the breathing pattern components to predict asthma control. However, more research involving different postures, such as supine or standing, is required to check maintenance of the identified association between asthma control and within-subject variability of breathing pattern components.

Some limitations underlie this research. We did not include patients with partially controlled asthma (ACQ7-item score between 0.75 and 1.50) so that ACQ7-item score could be used as a binary outcome within the recruited sample. A causal or coincidental relationship between within-subject variability and asthma control could not be determined from our findings due to the selected study design. It is not known whether uncontrolled asthma preceded the increased within-subject variability of the breathing pattern components, or vice versa. However, we speculate that increased within-subject variability in the presence of uncontrolled asthma is likely to be the result of several changes of the respiratory system as previously proposed in the literature [26]. For example, dysfunctional breathing has been characterised as a change in the biomechanical and physiological components of breathing, resulting in intermittent or chronic respiratory symptoms, which can worsen asthma progress [26]. In any way, a future prospective cohort study is required to examine the exact nature of the relationship between the changes in quantifiable breathing pattern components and asthma control.

## Conclusion

The study showed that within-subject variability of timing parameters and TA motion predicts and classifies levels of asthma control, but same results were not found for mean values of them. It is concluded that increased within-subject variability of RR, Ti/Te and RCampe/ABampe is associated with uncontrolled asthma shedding a light on the clinical importance of the changes in tidal breathing regularity as an adjunct physiological marker of asthma control.

## Data Availability

The datasets used and analysed during the current study are available from the corresponding author on reasonable request.

## Abbreviations

TA: Thoracoabdominal
ACQ7-item: Asthma Control Questionnaire
RIP: Respiratory Inductive Plethysmography
RR: Respiratory Rate
Ti/Te: Ratio of inspiration phase over expiration phase
RCampe/ABampe: Ratio of ribcage amplitude to abdominal amplitude during the expiration phase
SLP: Structured Light Plethysmography
CoV%: Coefficient of Variance expressed in a percentage
DB: Dysfunctional breathing
NQ: Nijmegen Questionnaire
sd: Standard deviation
ROC: Receiver Operating Characteristic curve
